# Bis(ethyl­enediamine-κ^2^ *N*,*N*′)bis­(methanol-κ*O*)copper(II) benzene-1,4-dicarboxyl­ate methanol disolvate

**DOI:** 10.1107/S1600536812028942

**Published:** 2012-06-30

**Authors:** Abolfazl Abbaszadeh, Nasser Safari, Vahid Amani, Behrouz Notash

**Affiliations:** a Department of Chemistry, Shahid Beheshti University, G. C., Evin, Tehran 1983963113, Iran

## Abstract

In the cation of the title compound, [Cu(C_2_H_8_N_2_)_2_(CH_3_OH)_2_](C_8_H_4_O_4_)·2CH_3_OH, the Cu^II^ atom lies on an inversion centre. The four N atoms of two ethyl­enediamine ligands around the Cu^II^ atom form the equatorial plane, while two methanol O atoms in the axial positions complete a Jahn–Teller distorted octa­hedral coordination. The benzene-1,4-dicarboxyl­ate anion is centrosymmetric. In the crystal, C—H⋯O, N—H⋯O and O—H⋯O hydrogen bonds link the cations, the anions and the methanol solvent mol­ecules.

## Related literature

For the role of copper compounds in biology, see: Kovala-Demertzi *et al.* (1997 ▶). For background to copper coordination polymers with carboxyl­ate ligands, see: Eddaoudi *et al.* (2001 ▶); Wen *et al.* (2005 ▶). For related structures with copper(II) and carboxyl­ate anions, see: Al-Hashemi *et al.* (2010*a* ▶,*b* ▶).
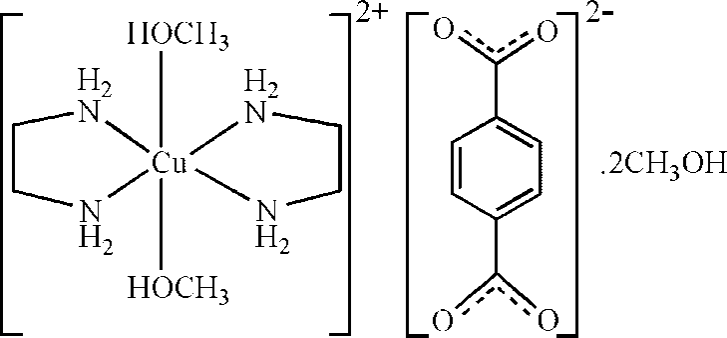


## Experimental

### 

#### Crystal data

[Cu(C_2_H_8_N_2_)_2_(CH_4_O)_2_](C_8_H_4_O_4_)·2CH_4_O
*M* *_r_* = 476.04
Monoclinic, 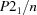

*a* = 7.3075 (15) Å
*b* = 12.416 (3) Å
*c* = 12.551 (3) Å
β = 92.43 (3)°
*V* = 1137.7 (5) Å^3^
*Z* = 2
Mo *K*α radiation
μ = 1.01 mm^−1^
*T* = 120 K
0.30 × 0.25 × 0.20 mm

#### Data collection

Stoe IPDS-2T diffractometer
Absorption correction: numerical (*X-SHAPE* and *X-RED*; Stoe & Cie, 2002 ▶) *T*_min_ = 0.752, *T*_max_ = 0.824
7838 measured reflections
3038 independent reflections
2347 reflections with *I* > 2σ(*I*)
*R*_int_ = 0.048

#### Refinement

*R*[*F* ^2^ > 2σ(*F* ^2^)] = 0.041
*wR*(*F* ^2^) = 0.085
*S* = 1.03
3038 reflections
159 parameters
2 restraints
H atoms treated by a mixture of independent and constrained refinement
Δρ_max_ = 0.34 e Å^−3^
Δρ_min_ = −0.31 e Å^−3^

### 

Data collection: *X-AREA* (Stoe & Cie, 2002 ▶); cell refinement: *X-AREA*; data reduction: *X-RED* (Stoe & Cie, 2002 ▶); program(s) used to solve structure: *SHELXS97* (Sheldrick, 2008 ▶); program(s) used to refine structure: *SHELXL97* (Sheldrick, 2008 ▶); molecular graphics: *ORTEP-3* (Farrugia, 1997 ▶); software used to prepare material for publication: *WinGX* (Farrugia, 1999 ▶).

## Supplementary Material

Crystal structure: contains datablock(s) I, global. DOI: 10.1107/S1600536812028942/hy2562sup1.cif

Structure factors: contains datablock(s) I. DOI: 10.1107/S1600536812028942/hy2562Isup2.hkl

Additional supplementary materials:  crystallographic information; 3D view; checkCIF report

## Figures and Tables

**Table 1:** Hydrogen-bond geometry (Å, °)

*D*—H⋯*A*	*D*—H	H⋯*A*	*D*⋯*A*	*D*—H⋯*A*
N1—H1*C*⋯O2	0.82 (3)	2.27 (3)	3.075 (3)	167 (3)
N1—H1*D*⋯O3^i^	0.84 (3)	2.20 (3)	3.009 (2)	162 (3)
N2—H2*C*⋯O3	0.86 (2)	2.12 (3)	2.976 (2)	169 (3)
N2—H2*D*⋯O1^i^	0.92 (2)	2.18 (3)	3.065 (3)	160 (2)
O3—H3*A*⋯O2	0.79 (2)	1.89 (2)	2.675 (2)	172 (3)
O4—H4*A*⋯O1^ii^	0.78 (2)	1.85 (2)	2.628 (2)	171 (3)
C7—H7*C*⋯O4^iii^	0.98	2.53	3.320 (3)	138

Symmetry codes: (i) 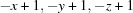; (ii) 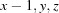; (iii) 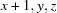.

**Fig. 1.**
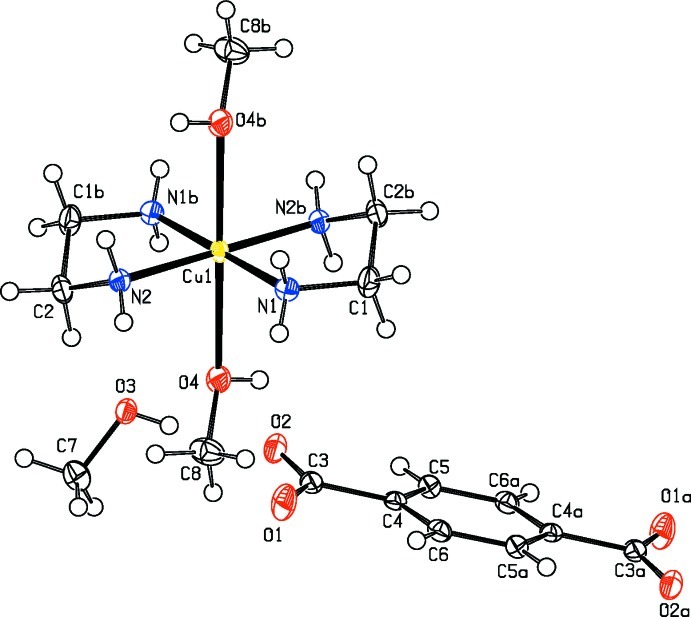
The molecular structure of the title compound with displacement ellipsoids drawn at the 50% probability level. [Symmetry codes: (a) 1-x, 2-y, 1-z; (b) -x, 1-y, 1-z.]

**Fig. 2.**
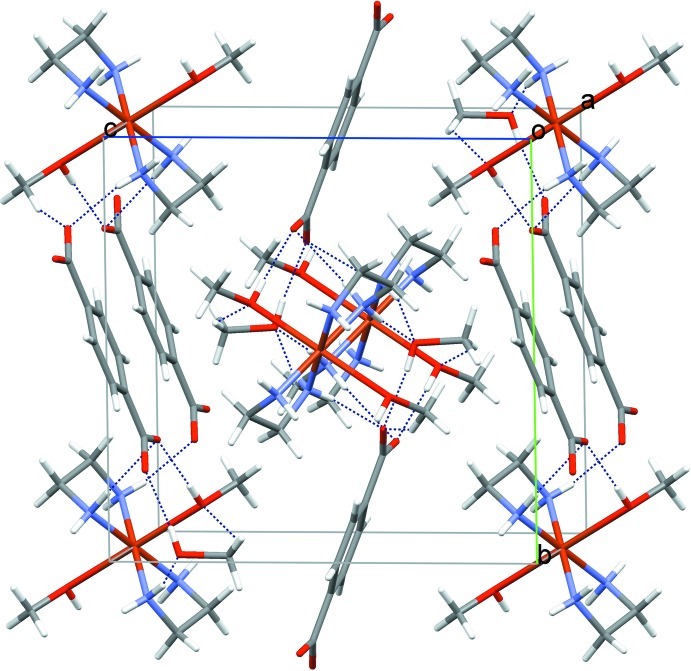
The packing diagram of the title compound showing hydrogen bonds as blue dashed lines.

## supplementary crystallographic information

### Comment

Copper plays a role in a number of biological processes with therapeutically administered drugs (Kovala-Demertzi *et al.*, 1997). Coordination chemistry of Cu(II) complexes is important as building-blocks to construct novel coordination architectures (Wen *et al.*, 2005). Carboxylate anions are widely used in the synthesis of coordination polymers (Eddaoudi *et al.*, 2001). In the recent years, we reported the synthesis and crystal structures of Cu(II) carboxylate complexes (Al-Hashemi *et al.*, 2010*a*,*b*). In order to expand this field, the title compound has been synthesized and its crystal structure is reported herein.

The asymmetric unit of the title compound (Fig. 1) consists a half of Cu^II^ ion, one ethylenediamine (en), one coordinated methanol, one uncoordinated methanol and a half of benzene-1,4-dicarboxylate anion. The Cu^II^ atom in the [Cu(en)_2_(CH_3_OH)_2_]^2+^ cation lies on an inversion centre. The four N atoms of the en ligands in the equatorial plane around the Cu^II^ atom form a slightly distorted square-planar arrangement, while the slightly distorted Jahn-Teller octahedral coordination is completed by two methanol O atoms in the axial positions. In the crystal, intermolecular C—H···O, N—H···O and O—H···O hydrogen bonds (Table 1) link the cations, the anions and the methanol solvent molecules (Fig. 2), which are effective in the stabilization of the structure.

### Experimental

Benzene-1,4-dicarboxylic acid (0.10 g, 0.59 mmol) was dissolved in 6 ml methanol and 3.9 ml ethylenediamine (0.30 mol *L*^-1^ in methanol). Then CuCl_2_.2H_2_O (0.10 g, 0.59 mmol) was added to the solution and the reaction mixture was stirred. After 10 min 2-methylimidazol (0.10 g, 1.18 mmol) was added to the stirred solution. The resulting violet solution stirred at 313 K for 25 min. This solution was left to evaporate slowly at room temperature. After one week, violet block crystals of the title compound were isolated (yield: 0.20 g, 70.2%).

### Refinement

H atoms bonded to O and N atoms were found in a difference Fourier map and refined isotropically. H atoms bonded to C atoms were positioned geometrically and refined as riding atoms, with C—H = 0.95 (aromatic), 0.99 (CH_2_) and 0.98 (CH_3_) Å and with *U*_iso_(H) = 1.2(1.5 for methyl)*U*_eq_(C).

### Crystal data

**Table:**

[Cu(C_2_H_8_N_2_)_2_(CH_4_O)_2_](C_8_H_4_O_4_)·2CH_4_O	*F*(000) = 506
*M**_r_* = 476.04	*D*_x_ = 1.390 Mg m^−^^3^
Monoclinic, *P*2_1_/*n*	Mo *K*α radiation, λ = 0.71073 Å
Hall symbol: -P 2yn	Cell parameters from 3038 reflections
*a* = 7.3075 (15) Å	θ = 3.2–29.1°
*b* = 12.416 (3) Å	µ = 1.01 mm^−^^1^
*c* = 12.551 (3) Å	*T* = 120 K
β = 92.43 (3)°	Block, violet
*V* = 1137.7 (5) Å^3^	0.30 × 0.25 × 0.20 mm
*Z* = 2	

### Data collection

**Table:**

Stoe IPDS-2T diffractometer	3038 independent reflections
Radiation source: fine-focus sealed tube	2347 reflections with *I* > 2σ(*I*)
Graphite monochromator	*R*_int_ = 0.048
ω scans	θ_max_ = 29.1°, θ_min_ = 3.2°
Absorption correction: numerical (*X-SHAPE* and *X-RED*; Stoe & Cie, 2002)	*h* = −9→10
*T*_min_ = 0.752, *T*_max_ = 0.824	*k* = −17→15
7838 measured reflections	*l* = −17→13

### Refinement

**Table:**

Refinement on *F*^2^	Primary atom site location: structure-invariant direct methods
Least-squares matrix: full	Secondary atom site location: difference Fourier map
*R*[*F*^2^ > 2σ(*F*^2^)] = 0.041	Hydrogen site location: inferred from neighbouring sites
*wR*(*F*^2^) = 0.085	H atoms treated by a mixture of independent and constrained refinement
*S* = 1.03	*w* = 1/[σ^2^(*F*_o_^2^) + (0.0404*P*)^2^ + 0.2093*P*] where *P* = (*F*_o_^2^ + 2*F*_c_^2^)/3
3038 reflections	(Δ/σ)_max_ = 0.001
159 parameters	Δρ_max_ = 0.34 e Å^−^^3^
2 restraints	Δρ_min_ = −0.31 e Å^−^^3^

### Special details

**Table:**

Geometry. All e.s.d.'s (except the e.s.d. in the dihedral angle between two l.s. planes) are estimated using the full covariance matrix. The cell e.s.d.'s are taken into account individually in the estimation of e.s.d.'s in distances, angles and torsion angles; correlations between e.s.d.'s in cell parameters are only used when they are defined by crystal symmetry. An approximate (isotropic) treatment of cell e.s.d.'s is used for estimating e.s.d.'s involving l.s. planes.
Refinement. Refinement of *F*^2^ against ALL reflections. The weighted *R*-factor *wR* and goodness of fit *S* are based on *F*^2^, conventional *R*-factors *R* are based on *F*, with *F* set to zero for negative *F*^2^. The threshold expression of *F*^2^ > σ(*F*^2^) is used only for calculating *R*-factors(gt) *etc*. and is not relevant to the choice of reflections for refinement. *R*-factors based on *F*^2^ are statistically about twice as large as those based on *F*, and *R*- factors based on ALL data will be even larger.

### Fractional atomic coordinates and isotropic or equivalent isotropic displacement parameters (Å^2^)

**Table:**

	*x*	*y*	*z*	*U*_iso_*/*U*_eq_	
Cu1	0.0000	0.5000	0.5000	0.01297 (9)	
O1	0.7634 (2)	0.76878 (13)	0.41449 (15)	0.0307 (4)	
O2	0.4701 (2)	0.71851 (12)	0.40999 (14)	0.0272 (4)	
O3	0.49935 (19)	0.51748 (11)	0.33602 (12)	0.0186 (3)	
O4	−0.0673 (2)	0.60364 (12)	0.33221 (13)	0.0214 (3)	
N1	0.1952 (2)	0.60613 (15)	0.55183 (16)	0.0171 (3)	
N2	0.1712 (2)	0.40775 (14)	0.41747 (15)	0.0168 (3)	
C1	0.1084 (3)	0.69471 (18)	0.6108 (2)	0.0239 (5)	
H1A	0.0700	0.7530	0.5609	0.029*	
H1B	0.1969	0.7249	0.6648	0.029*	
C2	0.0570 (3)	0.35020 (18)	0.33503 (19)	0.0231 (4)	
H2A	0.1279	0.2910	0.3037	0.028*	
H2B	0.0174	0.4004	0.2772	0.028*	
C3	0.5963 (3)	0.78604 (16)	0.42582 (17)	0.0192 (4)	
C4	0.5456 (3)	0.89742 (16)	0.46348 (16)	0.0164 (4)	
C5	0.3620 (3)	0.92725 (17)	0.47038 (17)	0.0172 (4)	
H5	0.2676	0.8778	0.4502	0.021*	
C6	0.6820 (3)	0.97086 (16)	0.49332 (17)	0.0173 (4)	
H6	0.8069	0.9510	0.4888	0.021*	
C7	0.5385 (3)	0.5129 (2)	0.22584 (18)	0.0257 (5)	
H7A	0.4517	0.5586	0.1849	0.039*	
H7B	0.5272	0.4384	0.2006	0.039*	
H7C	0.6635	0.5386	0.2162	0.039*	
C8	0.0619 (3)	0.6480 (2)	0.2634 (2)	0.0299 (5)	
H8A	−0.0026	0.6885	0.2062	0.045*	
H8B	0.1329	0.5898	0.2324	0.045*	
H8C	0.1446	0.6964	0.3040	0.045*	
H3A	0.500 (4)	0.5781 (16)	0.355 (2)	0.036 (8)*	
H4A	−0.127 (4)	0.6514 (19)	0.353 (2)	0.040 (9)*	
H1C	0.258 (4)	0.632 (2)	0.506 (2)	0.025 (7)*	
H2C	0.256 (3)	0.445 (2)	0.389 (2)	0.026 (7)*	
H1D	0.266 (4)	0.573 (2)	0.596 (2)	0.029 (7)*	
H2D	0.220 (3)	0.358 (2)	0.465 (2)	0.026 (7)*	

### Atomic displacement parameters (Å^2^)

**Table:**

	*U*^11^	*U*^22^	*U*^33^	*U*^12^	*U*^13^	*U*^23^
Cu1	0.01172 (14)	0.01237 (14)	0.01498 (16)	0.00083 (14)	0.00256 (10)	−0.00271 (16)
O1	0.0276 (8)	0.0218 (8)	0.0430 (11)	0.0109 (6)	0.0046 (7)	−0.0054 (8)
O2	0.0341 (8)	0.0163 (7)	0.0319 (9)	−0.0010 (6)	0.0104 (7)	−0.0046 (7)
O3	0.0202 (6)	0.0142 (8)	0.0216 (7)	−0.0017 (5)	0.0041 (5)	−0.0032 (6)
O4	0.0197 (7)	0.0191 (7)	0.0261 (8)	0.0041 (6)	0.0064 (6)	−0.0008 (7)
N1	0.0154 (8)	0.0178 (8)	0.0180 (9)	0.0000 (6)	0.0009 (7)	−0.0001 (7)
N2	0.0179 (8)	0.0154 (8)	0.0175 (9)	0.0011 (6)	0.0037 (7)	−0.0013 (7)
C1	0.0232 (10)	0.0184 (10)	0.0306 (12)	−0.0037 (8)	0.0043 (9)	−0.0086 (9)
C2	0.0278 (11)	0.0210 (10)	0.0208 (11)	−0.0013 (8)	0.0071 (8)	−0.0074 (9)
C3	0.0267 (10)	0.0167 (9)	0.0145 (10)	0.0065 (8)	0.0031 (7)	0.0025 (8)
C4	0.0221 (9)	0.0154 (9)	0.0120 (9)	0.0038 (7)	0.0035 (7)	0.0036 (8)
C5	0.0198 (9)	0.0168 (9)	0.0152 (10)	0.0007 (7)	0.0014 (7)	−0.0007 (8)
C6	0.0179 (9)	0.0199 (10)	0.0140 (9)	0.0044 (7)	0.0019 (7)	0.0018 (7)
C7	0.0260 (9)	0.0297 (13)	0.0215 (10)	−0.0006 (9)	0.0006 (7)	−0.0047 (10)
C8	0.0245 (11)	0.0382 (13)	0.0274 (13)	0.0068 (9)	0.0077 (9)	0.0074 (11)

### Geometric parameters (Å, º)

**Table:**

Cu1—N2	2.0150 (17)	C1—H1B	0.9900
Cu1—N1	2.0286 (18)	C2—C1^i^	1.518 (3)
Cu1—O4	2.4990 (17)	C2—H2A	0.9900
O1—C3	1.254 (3)	C2—H2B	0.9900
O2—C3	1.256 (3)	C3—C4	1.513 (3)
O3—C7	1.425 (3)	C4—C6	1.391 (3)
O3—H3A	0.79 (2)	C4—C5	1.397 (3)
O4—C8	1.418 (3)	C5—C6^ii^	1.387 (3)
O4—H4A	0.78 (2)	C5—H5	0.9500
N1—C1	1.483 (3)	C6—C5^ii^	1.387 (3)
N1—H1C	0.82 (3)	C6—H6	0.9500
N1—H1D	0.84 (3)	C7—H7A	0.9800
N2—C2	1.485 (3)	C7—H7B	0.9800
N2—H2C	0.86 (2)	C7—H7C	0.9800
N2—H2D	0.92 (2)	C8—H8A	0.9800
C1—C2^i^	1.518 (3)	C8—H8B	0.9800
C1—H1A	0.9900	C8—H8C	0.9800
			
N2—Cu1—N2^i^	180.0	N2—C2—H2A	110.2
N2—Cu1—N1	95.19 (7)	C1^i^—C2—H2A	110.2
N2^i^—Cu1—N1	84.81 (7)	N2—C2—H2B	110.2
N1^i^—Cu1—N1	180.0	C1^i^—C2—H2B	110.2
O4—Cu1—N1	92.66 (7)	H2A—C2—H2B	108.5
O4—Cu1—N1^i^	87.34 (7)	O1—C3—O2	125.47 (19)
O4—Cu1—N2	87.92 (7)	O1—C3—C4	116.41 (19)
O4—Cu1—N2^i^	92.08 (7)	O2—C3—C4	118.12 (18)
C7—O3—H3A	109 (2)	C6—C4—C5	119.24 (18)
C8—O4—H4A	107 (2)	C6—C4—C3	120.07 (18)
C1—N1—Cu1	109.39 (12)	C5—C4—C3	120.69 (18)
C1—N1—H1C	109.0 (19)	C6^ii^—C5—C4	119.91 (18)
Cu1—N1—H1C	116.0 (19)	C6^ii^—C5—H5	120.0
C1—N1—H1D	107 (2)	C4—C5—H5	120.0
Cu1—N1—H1D	107.2 (19)	C5^ii^—C6—C4	120.86 (18)
H1C—N1—H1D	108 (3)	C5^ii^—C6—H6	119.6
C2—N2—Cu1	106.80 (12)	C4—C6—H6	119.6
C2—N2—H2C	111.4 (18)	O3—C7—H7A	109.5
Cu1—N2—H2C	112.3 (19)	O3—C7—H7B	109.5
C2—N2—H2D	108.3 (17)	H7A—C7—H7B	109.5
Cu1—N2—H2D	106.7 (17)	O3—C7—H7C	109.5
H2C—N2—H2D	111 (2)	H7A—C7—H7C	109.5
N1—C1—C2^i^	108.47 (17)	H7B—C7—H7C	109.5
N1—C1—H1A	110.0	O4—C8—H8A	109.5
C2^i^—C1—H1A	110.0	O4—C8—H8B	109.5
N1—C1—H1B	110.0	H8A—C8—H8B	109.5
C2^i^—C1—H1B	110.0	O4—C8—H8C	109.5
H1A—C1—H1B	108.4	H8A—C8—H8C	109.5
N2—C2—C1^i^	107.42 (18)	H8B—C8—H8C	109.5
			
N2—Cu1—N1—C1	175.14 (15)	O2—C3—C4—C6	172.9 (2)
N2^i^—Cu1—N1—C1	−4.86 (15)	O1—C3—C4—C5	174.9 (2)
N1^i^—Cu1—N2—C2	23.15 (14)	O2—C3—C4—C5	−6.1 (3)
N1—Cu1—N2—C2	−156.85 (14)	C6—C4—C5—C6^ii^	0.0 (3)
Cu1—N1—C1—C2^i^	31.5 (2)	C3—C4—C5—C6^ii^	178.98 (19)
Cu1—N2—C2—C1^i^	−46.2 (2)	C5—C4—C6—C5^ii^	0.0 (3)
O1—C3—C4—C6	−6.2 (3)	C3—C4—C6—C5^ii^	−178.99 (19)

Symmetry codes: (i) −*x*, −*y*+1, −*z*+1; (ii) −*x*+1, −*y*+2, −*z*+1.

### Hydrogen-bond geometry (Å, º)

**Table:**

*D*—H···*A*	*D*—H	H···*A*	*D*···*A*	*D*—H···*A*
N1—H1*C*···O2	0.82 (3)	2.27 (3)	3.075 (3)	167 (3)
N1—H1*D*···O3^iii^	0.84 (3)	2.20 (3)	3.009 (2)	162 (3)
N2—H2*C*···O3	0.86 (2)	2.12 (3)	2.976 (2)	169 (3)
N2—H2*D*···O1^iii^	0.92 (2)	2.18 (3)	3.065 (3)	160 (2)
O3—H3*A*···O2	0.79 (2)	1.89 (2)	2.675 (2)	172 (3)
O4—H4*A*···O1^iv^	0.78 (2)	1.85 (2)	2.628 (2)	171 (3)
C7—H7*C*···O4^v^	0.98	2.53	3.320 (3)	138

Symmetry codes: (iii) −*x*+1, −*y*+1, −*z*+1; (iv) *x*−1, *y*, *z*; (v) *x*+1, *y*, *z*.
